# Insecticidal Effects of Hemocoelic Delivery of *Bacillus thuringiensis* Cry Toxins in *Achaea janata* Larvae

**DOI:** 10.3389/fphys.2017.00289

**Published:** 2017-05-10

**Authors:** Thuirei J. Ningshen, Vinod K. Chauhan, Narender K. Dhania, Aparna Dutta-Gupta

**Affiliations:** Department of Animal Biology, School of Life Sciences, University of HyderabadHyderabad, India

**Keywords:** *Achaea janata*, *Bacillus thuringiensis*, cry toxins, fat body, hemocoelic injection

## Abstract

Insecticidal effects of *Bacillus thuringiensis* Cry toxins in hemocoel of larvae have not been properly evaluated. In the present study, hemocoelic injection of four representative Cry toxins i.e., Cry1Aa, Cry1Ab, Cry1Ac, and DOR5 to an economically important lepidopteran insect pest *Achaea janata*, induced larval mortality, reduced larval growth rate and gave rise to smaller pupae, all in a dose-dependent manner. We observed extensive degeneration as well as the disintegration of larval tissues, most notably, fat body, and the possible involvement of lysosomal enzymes in tissue histolysis. The resultant “hypoproteinemia” and most relevantly, the drastic reduction of 80–85 kDa hexamerin proteins levels of hemolymph could be attributed to the pathological state of the fat body induced by Cry toxin injection. Formation of non-viable larval-pupal intermediates and emergence of defective adults also indicate toxicity effects of Cry toxins during metamorphosis. Thus, findings from our study suggest Cry toxins in larval hemocoel are also toxic to *A. janata* larval survival and subsequent development.

## Introduction

Insecticidal proteins called crystal (Cry) toxins, produced as crystal inclusions by gram-positive bacteria *Bacillus thuringiensis* (Bt), are toxic to a wide range of insects including those belonging to the order Lepidoptera (Bravo et al., 2011). When susceptible insects ingest the Cry toxins, they are solubilized in the alkaline medium of the gut, processed by the gut proteases to yield the active toxins which then bind to specific receptors located on the brush-border membranes of the larval midgut epithelial cells, followed by insertion into the cell membrane, leading to pore formation, then cell lysis, and eventual death of the insects (Ferré and Van Rie, 2002; Bravo et al., 2007). Development of Bt-based sprayable products and Bt transgenic crops have provided valuable alternatives to chemical insecticides as they are more insect species-specific and least harmful to the natural enemies (James, 2009; Bravo et al., 2011). However, as a consequence of the uncontrolled widespread use of Bt-based biopesticides in agriculture, a number of insect species including those belonging to Lepidoptera have developed resistance to Bt Cry toxins (Ferré and Van Rie, 2002; Tabashnik et al., 2005, 2013). Further, laboratory-selected resistance experiments have indicated the potential of many species to still evolve resistance against Cry toxins (Bravo et al., 2011). As an effort to tackle this problem, many workers have been continuously working to understand various aspects of modes of action and mechanisms of pest's resistance to Cry toxins after oral ingestion (Bravo and Soberón, 2008; Pardo-López et al., 2009), but no effective control strategy has been developed to date.

Earlier studies have suggested a possible mechanism where Cry toxins ingested along with the feed can also penetrate from gut through the gut wall to hemolymph in the insect hemocoel, which accumulate in the insect body and suppress the reproductive potential (Arpaia et al., 2000; Hussein et al., 2006). The fat body of lepidopteran larvae is the principal metabolic center, synthesizes, and releases a large number of haemolymph proteins including hexamerins, which play an important role during the pupal-adult transformation (Kanost et al., 1990). A recent report in *Helicoverpa armigera* further reveals accumulation of toxin in fat body and hemolymph of larvae fed with artificial diet containing high dosage of Cry1Ac (Zhao et al., 2016). Hemocoelic injection of Cry1 toxins into *Lymantria dispar* (Lepidoptera) and *Neobellieria bullata* (Diptera) larvae was found to illicit various toxic responses (Cerstians et al., 2001). However, proper evaluation of insecticidal effects of Cry toxins in hemocoelic visceral organs of insects has been lacking. Hence, the present study was undertaken to generate useful information on various aspects of toxicity induced by Cry toxins in the larvae of an economically important lepidopteran pest *Achaea janata*. Among various insect pests of castor, *A. janata* is a major pest in Indian subcontinent, whose larvae feed on castor foliage leading to serious crop loss. Hemocoelic injection of four representative Cry toxins i.e., Cry1Aa, Cry1Ab, Cry1Ac, and DOR5 to *A. janata* larvae, induced mortality, reduced growth rate and gave rise to smaller pupae, all in a dose-dependent manner. We also observed defective metamorphosis during larval-pupal and pupal-adult transformation. As we earlier reported the presence of Cry toxin binding protein (Aminopeptidase-N) in larval fat body of *A. janata* (Budatha et al., 2007), an attempt was also made to evaluate the effect of Cry toxin on the haemolymph protein profile. The study suggests Cry toxins in hemocoel are insecticidal to larval forms of *A. janata*, have deleterious effects on larval survival, growth, and subsequent development.

## Materials and methods

### Insect culture

*Achaea janata* egg masses collected from castor fields of Indian Institute of Oilseeds Research, Hyderabad were allowed to hatch into neonate larvae which were then reared on fresh castor leaves (*Ricinus communis*) as diet, at 27 ± 2°C in the insect culture facility under a photoperiod of 14:10 h (light:dark) and 60–70% relative humidity. The larval development proceeds through five instars and the whole life cycle is completed in about 35–40 days. Each instar from first to fourth lasts for about 2 days while the final or fifth instar lasts 4–5 days. The fifth instar is further classified into early (5E) and late (5L) fifth instar which lasts 2 and 2–3 days, respectively, then followed by non-feeding pre-pupal (PP) stage.

### Feeding bioassay

Feeding bioassay was performed on castor leave discs. Both sides of the leave discs were painted with Cry toxins, shade dried and placed onto moist cotton in petriplates. Seven treatment concentrations i.e., 2, 4, 6, 8, 10, 12, and 14 ng/cm^2^ and one control [carrier solvent coated; insect Ringer containing 1 mM of Tris-HCl (pH 7.0)] were selected. Triplicates were maintained for each treatment and twenty 2 days old *A. janata* larvae were released on each leaf disc. Mortality was recorded every 24 h interval for 5 days and LC_50_ was calculated with 2 days post-treatment data using probit analysis with the help of Finney's table (Finney, 1952).

### Collection of larval tissues

Larvae were narcotized on ice and tissues were dissected out in ice-cold insect Ringer solution (130 mM NaCl, 0.5 mM KCl, and 0.1 mM CaCl_2_) and used immediately. A cut was made in the ventral region through full length of larvae, along with midgut, perivisceral fat body, Malpighian tubule, and salivary gland were dissected out. Further, experiments were carried out using these tissue.

### Preparation of activated cry toxins

The DOR5 Bt spores and crystals were obtained using the method described by Aronson et al. (1971). Spores and crystals were separated by differential ultracentrifugation on a discontinuous sucrose density gradient in a Beckman L8–80M ultracentrifuge with a SW 50.1 rotor operating at 42,000 rpm for 4 h at 4°C (Thomas and Ellar, 1983). On the other hand, Cry1Aa, Cry1Ab, and Cry1Ac protoxins were obtained from recombinant *Escherichia coli* JM103 strains ECE52, ECE53, and ECE54 harboring *cry1Aa, cry1Ab*, and *cry1Ac* genes respectively, which were supplied by *Bacillus* Genetic Stock Center (Ohio State University, USA). The activated toxins were prepared according to the method of Lee et al. (1992) and purified by gel filtration on Sephadex G-100 column.

### Hemocoelic cry toxin injection assay

Third instar larvae were narcotized on ice for 15 min and were then injected individually with activated Cry1Aa, Cry1Ab, Cry1Ac, and DOR5 (Directorate of Oilseeds Research Strain 5) Cry toxin doses of 10, 50, 100, 150, and 200 ng/0.2 g body weight through the mid-dorsal region using a Hamilton microsyringe. Control larvae received the same volume of the carrier solvent. Immediately after injection, the wound was sealed with bee wax and the larvae were again placed on ice for 15 min before transferring them back to the rearing chamber provided with fresh castor leaves. A minimum of 20 larvae were/was used for each dose and the experiment was repeated three times independently. The body weight of larva was examined every 24 h for 5 days. Larval mortality was recorded 2 days post-injection (dpi). The weights of resultant pupae from toxin injected larvae and control were also recorded.

### Western blotting

Hemolymph from required larval stages were collected in centrifuge tubes precoated with 0.025% phenylthiourea in insect Ringer solution by cutting the proleg and were then centrifuged at 1000 × g for 3 min at 4°C to sediment the hemocytes. The supernatant was diluted with 10 mM Tris-HCl (pH 7.8) and used immediately. Total protein content was estimated by Bradford's method (1976) using BSA as standard. The hemolymph proteins were resolved by 10% SDS-PAGE and electro-blotted onto a nitrocellulose membrane using Trans-Blot apparatus (Bio-Rad) according to the procedure of Towbin et al. (1979). Non-specific binding sites were blocked with 5% skimmed milk (w/v) in Tris-buffered saline (TBS) and then incubated with *A. janata* hexamerin polyclonal antibody (1:5,000 dilutions) (Budatha et al., 2011). Subsequently, the blots were incubated with ALP-conjugated goat anti-rabbit IgG and finally detected with NBT-BCIP substrate (Sigma-Aldrich).

### Hematoxylin-eosin (HE) staining

Two days post-injection, various larval tissues were dissected out in ice-cold Ringer solution and then fixed in 4% paraformaldehyde. After washing with 0.1 M phosphate buffered saline (PBS, pH 7.4), the tissues were dehydrated in ethanol series and embedded in paraffin. Sections of 5 μm thickness were prepared using rotary microtome (Leica). The sections were deparafinized in xylene, rehydrated in ethanol series, followed by staining with hematoxylin (nuclear stain) and eosin (cytoplasmic stain). The sections were then dehydrated in ethanol series, cleared in xylene and mounted using DPX mountant.

### Acid phosphatase (ACP) assay

Acid phosphatase assay is widely used as the lysosomal marker for the demonstration of tissue degeneration and remodeling in various insect models. The assay was carried out according to the method of Henrikson and Clever (1972) with minor modifications. The reaction mixture containing 100 mM sodium acetate buffer (pH 5.0) and protein preparation (100 μg) was incubated at 37°C for 10 min to exclude glucose-6-phosphatase activity. The reaction was initiated with the addition of 8 mM *p*-nitrophenyl bi-sodium phosphate followed by incubation for 1 h at 37°C. The reaction was terminated by adding 0.5 ml of 0.1 N NaOH. The yellow color thus developed was measured at 410 nm against a substrate blank. The *p*-nitrophenol (PNP) was used for the preparation of a standard curve. The activity of the enzyme was expressed as nanomoles of PNP released/h/μg protein. The assay was performed with 2 days post-injected larval tissues.

### Phenotypic analysis

This was primarily carried out to analyze the effect of Cry toxin on the gross morphology of the insect. Photographs were taken with a FinePix S9600 digital camera (Nikon). The wings of the insects were placed on aluminum stubs and coated with gold in a FullamEMS-76 m evaporator for 15 min and the wing scale morphology was examined using Jeol scanning electron microscope.

### Statistical analysis

Data are expressed as mean ± standard deviation of three independent experiments (*n* = 3). Differences between groups were analyzed for statistical significance by One-Way ANOVA followed by Students-Newman-Keuls (SNK) test using SigmaPlot 11.0 software. A probability of *p* < 0.05 is considered statistically significant.

## Results

### Toxicity effects of cry toxin injection in *A. janata*

Cry1Aa, Cry1Ab, Cry1Ac, and DOR5 Cry protoxins and their subsequent activated forms were prepared and then purified by gel filtration using Sephadex G-100 column (Supplementary Figure 1). Lepidopteran-specific insecticidal gene profiling of DOR5 Bt isolate revealed the presence *cry1Aa, cry1Ab, cry1C, cry2A, cry2B*, and *vip* (vegetative insecticidal proteins) genes (unpublished data).

**Figure 1:**
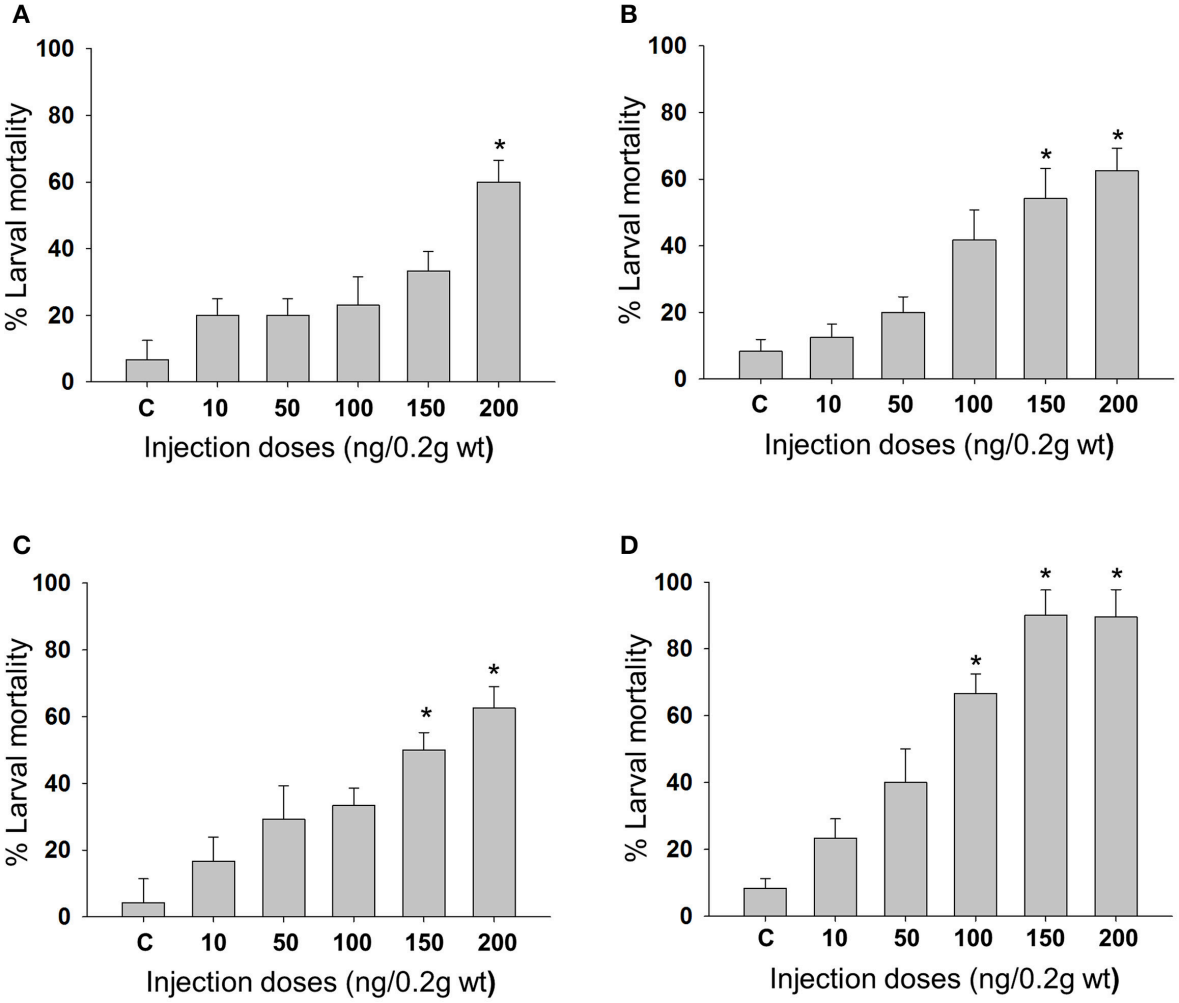
**Mortality of ***A. janata*** larvae upon Cry toxin injection**. Third instar larvae were injected with Cry toxin doses of 10, 50, 100, 150, and 200 ng/0.2 g body weight. Percentage of larval mortality for each dose was calculated 2 days post-injection. **(A)** Cry1Aa; **(B)** Cry1Ab; **(C)** Cry1Ac; **(D)** DOR5. Values presented are the mean ± standard deviation of three independent experiments (*n* = 3). ^*^represent ≥ 50% mortality.

**Figure 2:**
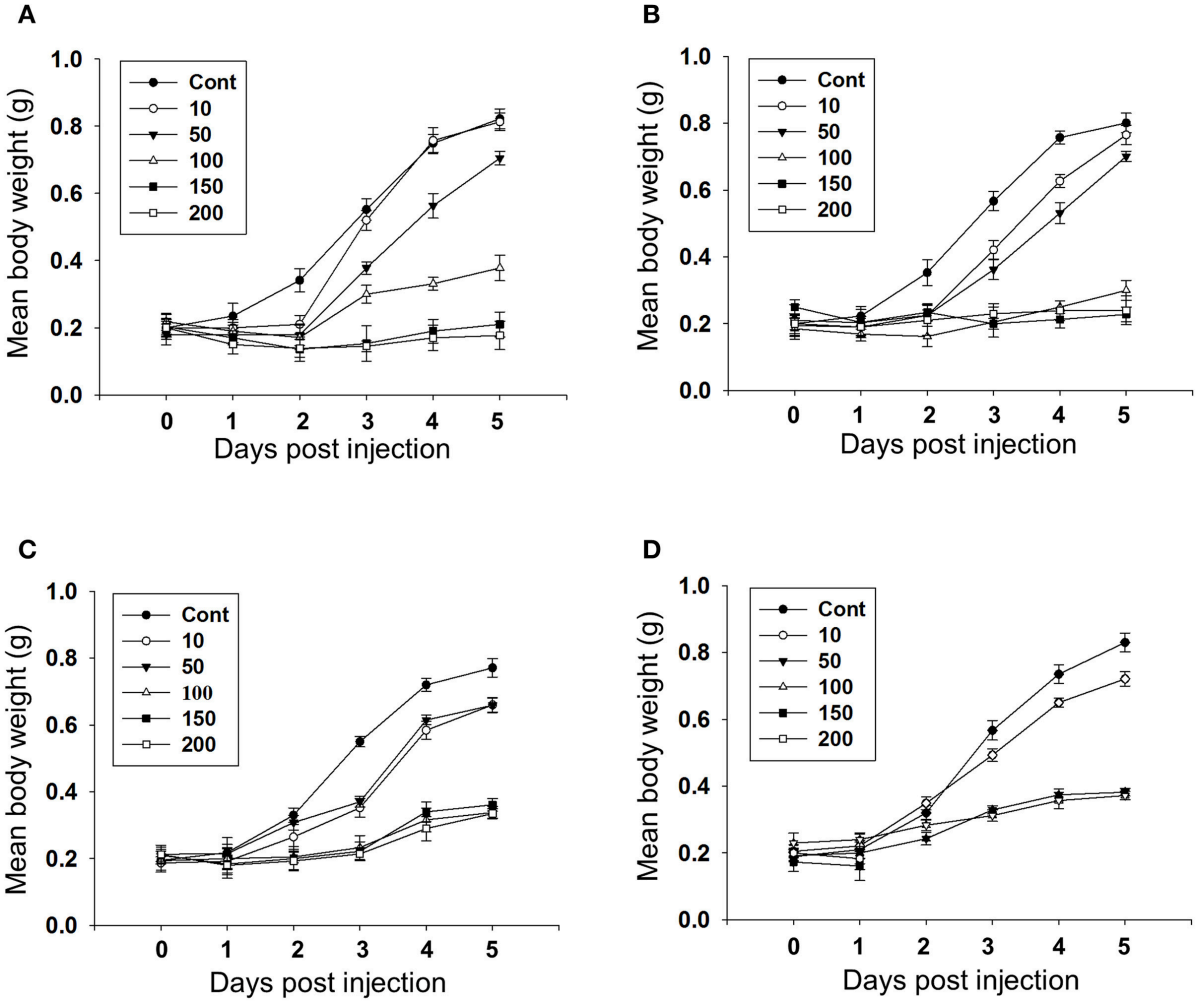
**Effect of Cry toxin injection on larval growth**. Third instar larvae were injected with Cry toxin doses of 10, 50, 100, 150, and 200 ng/0.2 g body weight and the weight of larvae was measured every 24 h for 5 days. **(A)** Cry1Aa; **(B)** Cry1Ab; **(C)** Cry1Ac; **(D)** DOR5. Values presented are the mean ± standard deviation of three independent experiments (*n* = 3).

**Figure 3:**
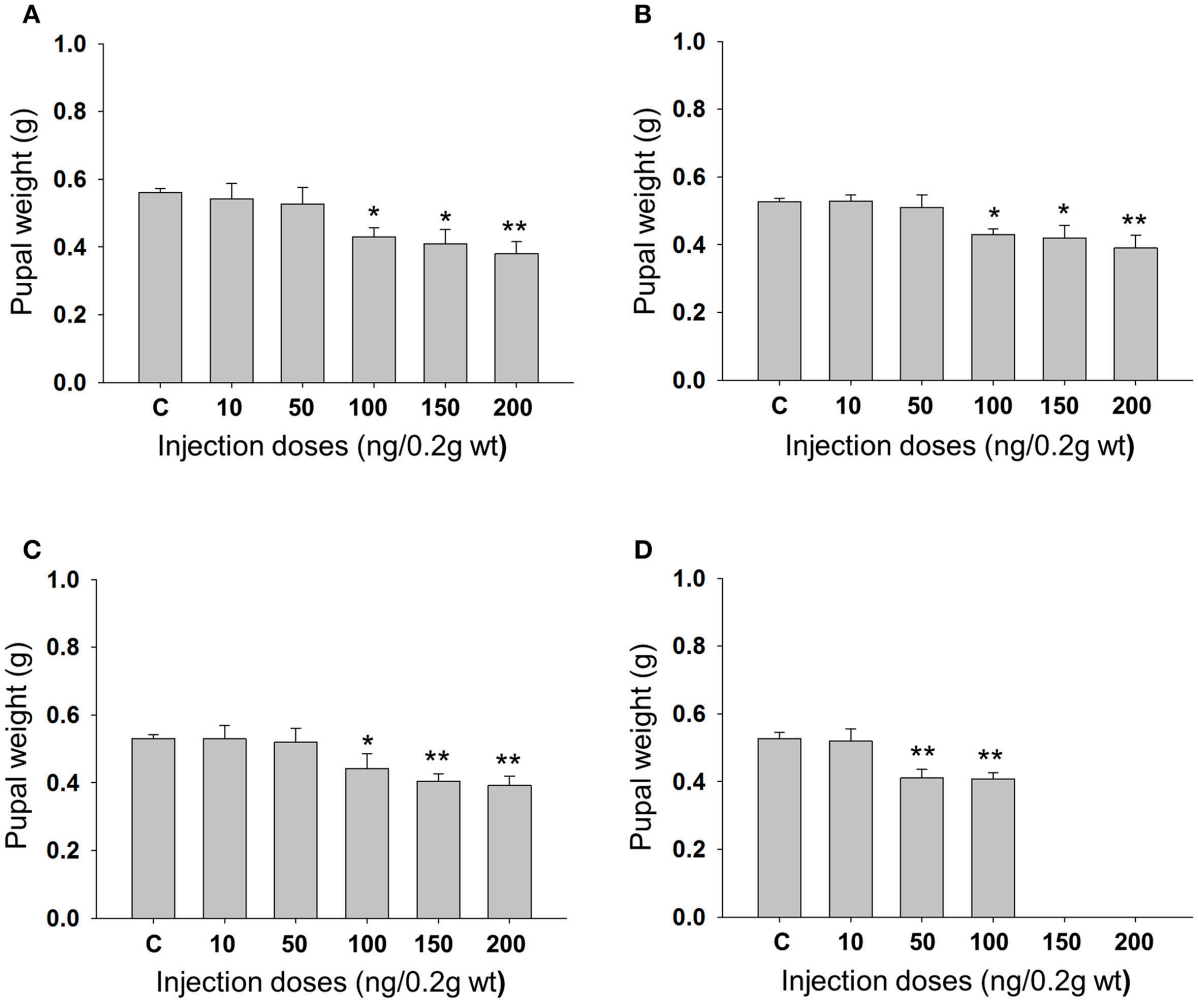
**Effect of larval Cry toxin injection on the pupal weight**. Third instar larvae were injected with Cry toxin doses of 10, 50, 100, 150, and 200 ng/0.2 g body weight. **(A)** Cry1Aa; **(B)** Cry1Ab; **(C)** Cry1Ac; **(D)** DOR5, they were allowed to feed, grow, and molt into pupae. Values presented are mean ± standard deviation of three independent experiments (*n* = 3). Significance between groups was tested by One-Way ANOVA followed by Student-Newman-Keuls (SNK) test using SigmaPlot 11.0 software. Means marked with ^*^p < 0.05, ^**^p < 0.001 indicate statistical significance.

Feeding bioassay with 2 days old *A. janata* larvae showed that recombinant Cry toxins i.e., Cry1Aa, Cry1Ab, and Cry1Ac feeding causes larval mortality, However, DOR5 Cry toxins showed much higher insecticidal activity with fairly low LC_50_ (4.9 ng/cm^2^ of leaf surface) (Table 1). Hemocoelic injection with doses of 200 ng/0.2 g body weight of Cry1Aa (Figure 1A), 150 ng/0.2 g body weight each of Cry1Ab (Figure 1B) and Cry1Ac (Figure 1C), and 100 ng/0.2 g body weight of DOR5 (Figure 1D) toxins resulted in 50–70% larval mortality. Further, increase in DOR 5 toxin dose to 150 ng/0.2 g body weight, caused nearly 100% larval mortality (Figure 1D). All the doses of Cry1Aa, Cry1Ab, Cry1Ac, and DOR5 Cry toxins reduced food consumption. The toxins inhibited larval growth in a dose-dependent manner. Results presented in Figure 2 clearly show that Cry1Aa (Figure 2A), Cry1Ab (Figure 2B), and Cry1Ac (Figure 2C) at doses ≥100 ng/0.2 g body weight reduced larval growth and the mean body weight was fairly low after 5 days after injection. Further, DOR5 Cry toxin was found to be more effective than the recombinant toxins (Figure 2D). The control larvae reached a maximum body weight of around 0.8 g during fifth instar, while the toxin injected larvae weighed around 0.3 g or less, even at 5 days post-injection (number of days after which the larvae should be in fifth instar). Furthermore, injection of ≥100 ng/0.2 g body weight of Cry1Aa (Figure 3A), Cry1Ab (Figure 3B), and Cry1Ac (Figure 3C) and ≥50 ng/0.2 g body weight of DOR5 (Figure 3D) toxins caused the significant reduction in the weight of resultant pupae (*p* < 0.05). The weight of the control pupa reached 0.55 ± 0.013 g while the pupae obtained after injection weighed only around 0.38 ± 0.04 g (Figure 3D).

**Table 1:** **Evaluation of insecticidal activity of Cry toxins against ***A. janata*** larvae after oral ingestion [Details of experiment are provided in Section Cross-Compression Entropy (CCE)]**.

**Cry toxin**	**Feeding bioassay LC_50_(ng/cm^2^ of leaf surface)**
Cry 1Aa	107.56
Cry1Ab	47.83
Cry1Ac	35.87
DOR5	4.908

### Pathological effects of cry toxin injection

**Figure 4:**
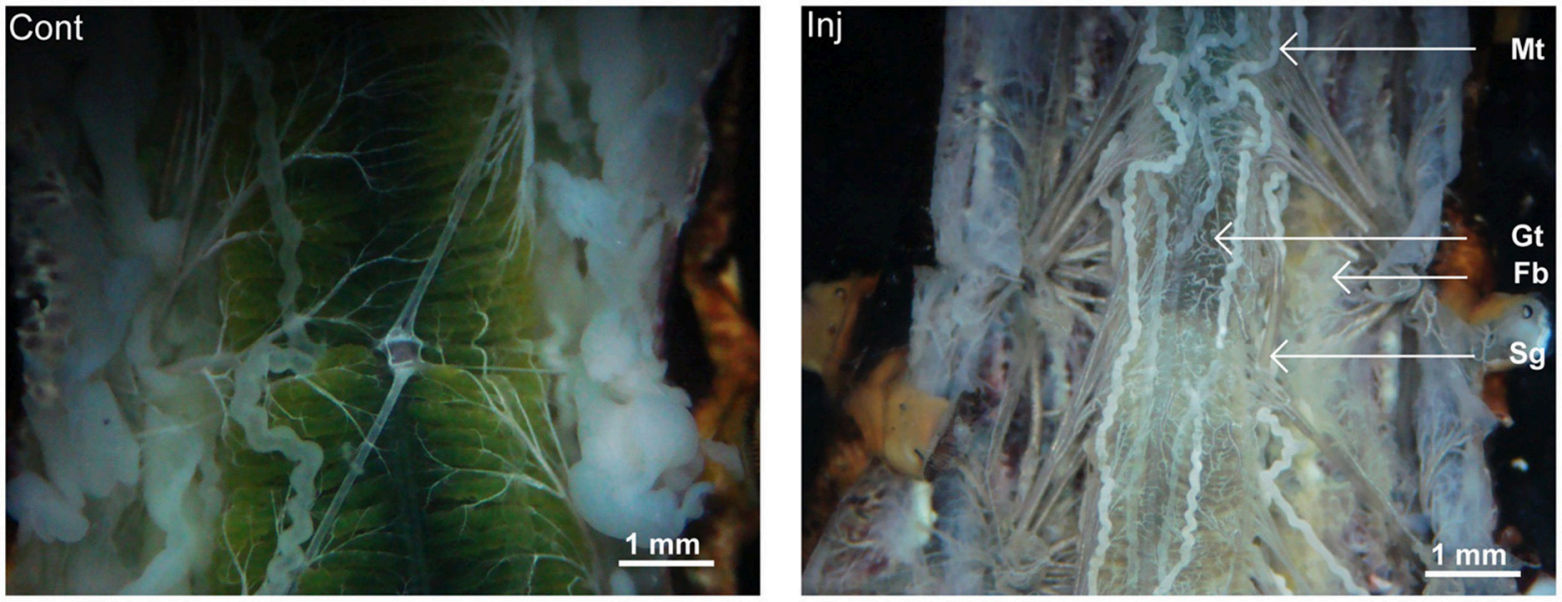
**Pathological condition of Cry toxin injected ***A. janata*** larvae**. Two days post-injected larvae were dissected and morphological features of various visceral tissue was viewed. Note the differences between the morphology of the organs in control and Cry toxin injected (Inj, arrow), Gt, gut; Fb, fat body; Mt, Malpighian tubule; and Sg, salivary gland.

Observation of vital tissues of 2 days post-injected larvae revealed thin and delicately developed fat body tissue (Figure 4). The midgut of the toxin injected larvae had comparatively narrower lumen with lower food contents and the Malpighian tubules appeared thin and opaque (Figure 4). The size of salivary glands of the toxin injected insects was much smaller (Figure 4).

**Figure 5:**
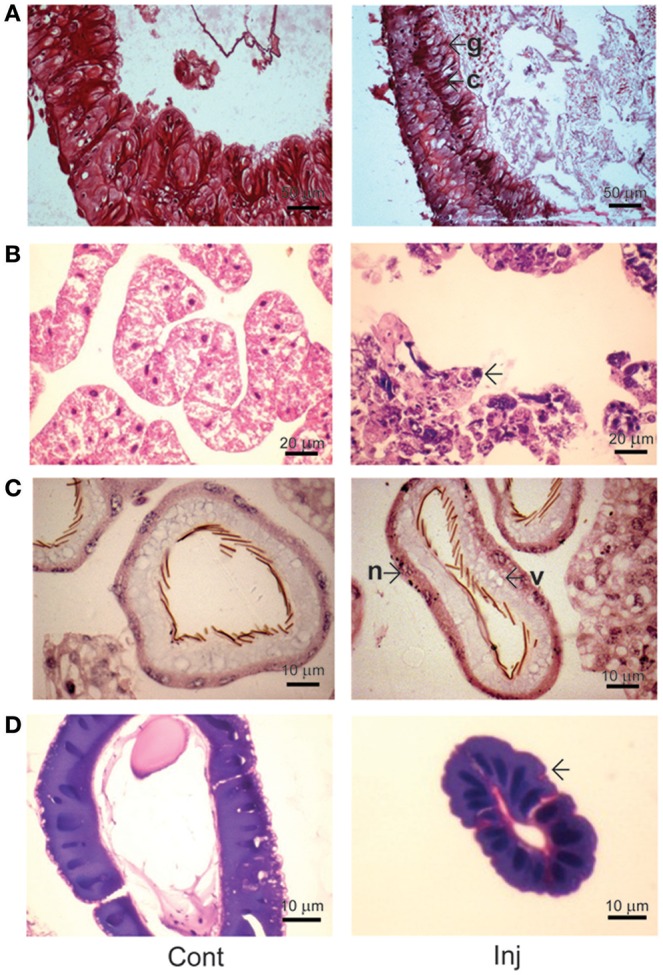
**Histological analysis of Cry toxin injected ***A. janata*** larval tissues**. Tissue sections were prepared from 2 days post-injected larvae and stained with hematoxylin-eosin (HE). **(A)** midgut; **(B)** fat body; **(C)** Malpighian tubule; **(D)** salivary gland. Note the cytological changes in tissue sections induced by Cry toxin injection (Inj, arrowhead), g, Goblet cell; c, columnar cell; v, vacuole; n, nucleus.

**Figure 6:**
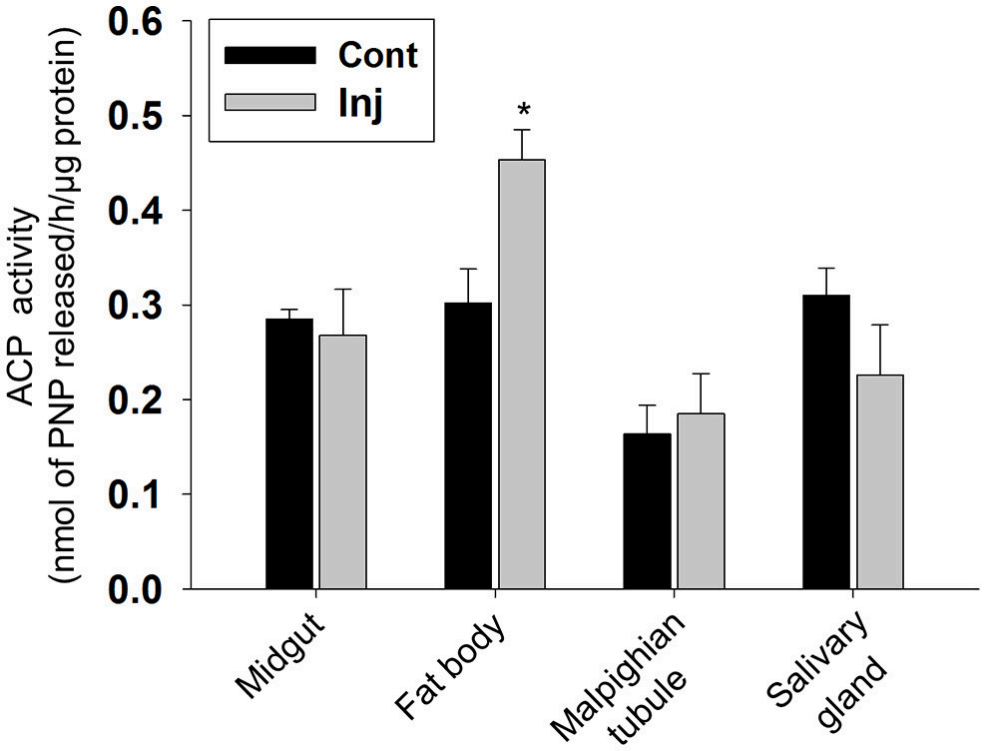
**Analysis of ACP activity in Cry toxin injected larval tissues**. Assay was performed with 2 days post-injected larval tissues. The enzyme activity is expressed as nmol of PNP released/h/mg protein. Values are the mean ± standard deviation of three independent experiments (*n* = 3). ^*^p < 0.050 (One-Way ANOVA followed by SNK-test).

Hematoxylin and eosin (HE) staining of midgut tissue sections of Cry toxin injected larvae revealed the presence of irregular arrangement of Goblet (g) as well as columnar (c) cells in the gut epithelium (Figure 5A; Inj). In fat body, extensive tissue degeneration was seen (Figure 5B; Inj), viability/non-viability of fat body tissue from control and toxin injected larvae was confirmed using propidium iodide and MTT [3-(4,5-dimethylthiazol-2-yl)-2,5-diphenyltetrazolium bromide] staining (Supplementary Figure 2). We observed vacuoles (v) in the cytoplasmic regions of the single cell layered epithelium of Malpighian tubule with chromatin condensation in the nucleus (n) of these cells (Figure 5C; Inj). The salivary gland of the Cry toxin injected larvae did not show major structural differences but condensation of nuclear chromatin and reduced diameter of the gland with much narrow lumen was observed (Figure 5D; Inj). HE staining revealed fat body to be most affected by haemocoelic Cry toxin injection. Estimation of ACP activity in tissues of Cry toxin injected larvae showed a significant (*p* < 0.05) increase in the level of enzyme activity only in fat body and not in other tissues (Figure 6).

### Effect of cry toxin injection on larval hemolymph hexamerin profile

**Figure 7:**
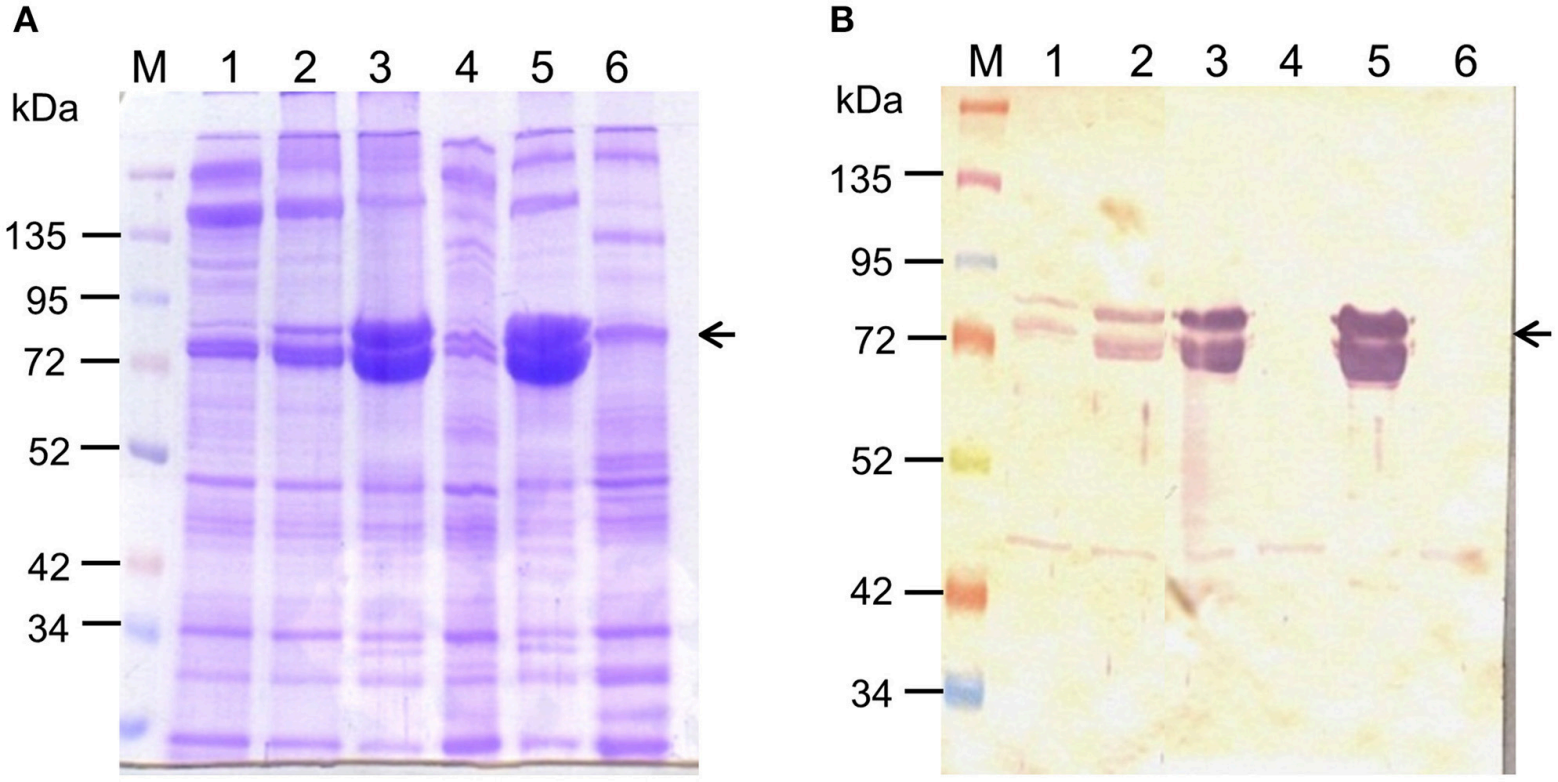
**Effect of Cry toxin injection on hemolymph hexamerin protein profile**. Hemolymph hexamerin proteins profile was analyzed 2 and 4 days post-injection. **(A)** 10% SDS-PAGE analysis of larval hemolymph proteins profile. **(B)** Western blot analysis of hexamerin protein profile. Note the disappearance of 80–85 kDa hexamerin protein from the hemolymph of 2 and 4 days post-injected larvae (arrowhead). lane M: protein ladder, lane 1: second instar (4 days old), lane 2: third instar (6 days old), lane 3: fourth instar control (2 dpi), lane 4: fourth instar Cry toxin injected (2 dpi), lane 5: fifth instar control (4 dpi), lane 6: fifth instar Cry toxin injected (4 days post-injection).

Upon injection of Cry toxins into third instar larvae, the increase in larval hemolymph total protein content (seen during normal larval development) was significantly inhibited (*p* < 0.001; Table 2). The total protein content of the 2 and 4 days post-injected control larvae reached 2.12 ± 0.06 and 2.3 ± 0.07 mg/mL hemolymph respectively, while the Cry toxin injected larvae of same chronological age showed only 0.3 ± 0.05 and 0.34 ± 0.04 mg/mL hemolymph respectively (Table 2). SDS-PAGE (Figure 7A) and western blot (Figure 7B) analyses revealed drastic reduction in 80–85 kDa hexamerin proteins level in hemolymph of the 2 (Figures 7A,B, Lane: 4) as well as 4 days post-injected (Figures 7A,B, Lane: 6) larvae. However, the hemolymph of 2 and 4 days post-injected control larvae showed fairly high amount of the 80–85 kDa hexamerin proteins (Figures 7A,B, Lanes: 3 and 5).

**Table 2:** **Effect of hemocoelic Cry toxin injection on larval hemolymph total protein content**.

	**2 dpi (4th instar) (±*SD*)**	**4 dpi (5th instar) (±*SD*)**
Control	2.12 ± 0.06	2.3 ± 0.07
Injected	0.3 ± 0.05^**^	0.34 ± 0.04^**^

*Total protein content expressed as mg/mL hemolymph*.

** *p < 0.01. Total protein content was analyzed 2 and 4 dpi of third instar larvae with Cry toxins. The results shown in the table represent DOR5 Cry toxin dose 100 ng/0.2 g body weight*.

### Effect of cry toxin injection on larval-pupal and pupal-adult transformation

**Figure 8:**
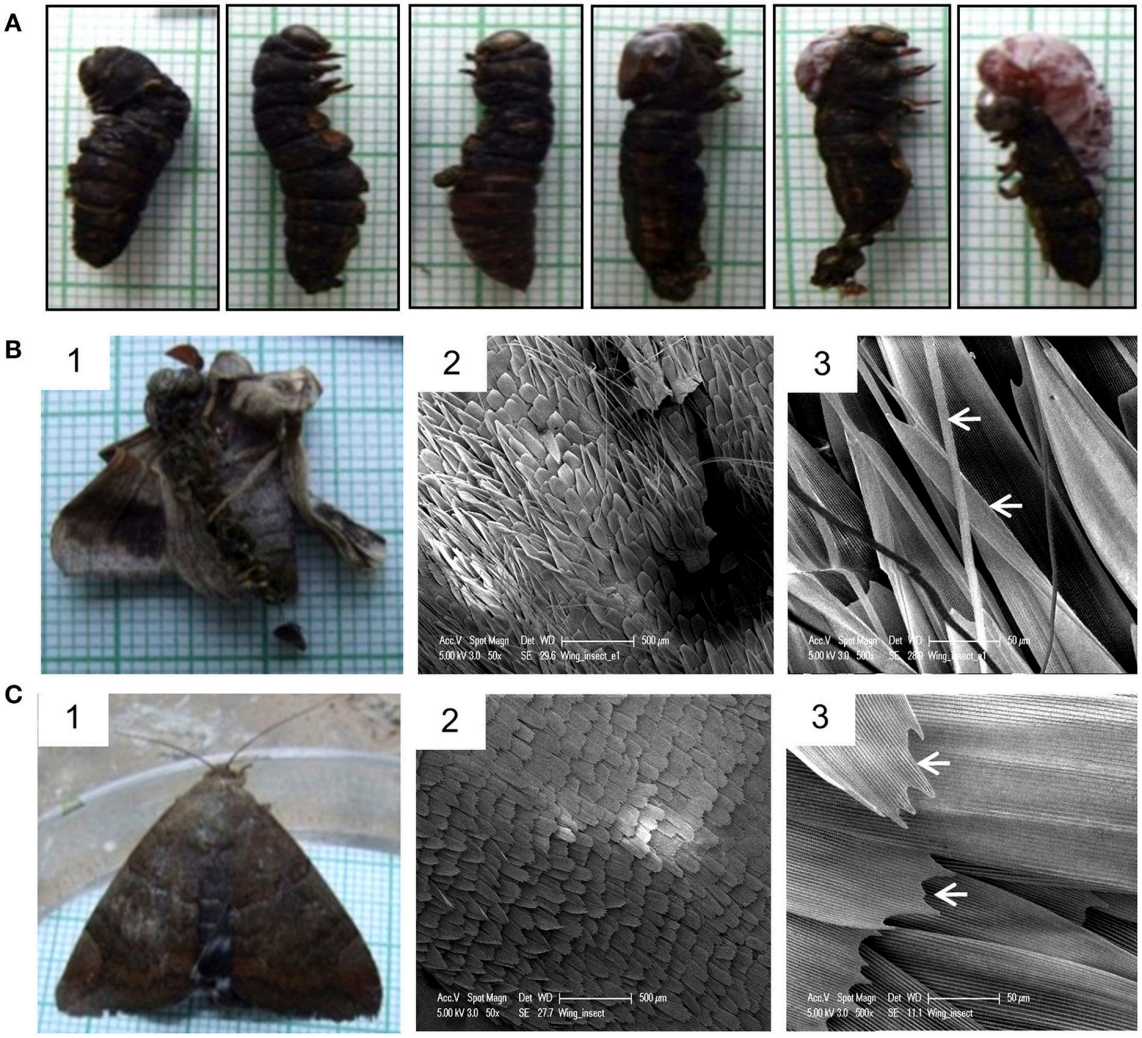
**Effect of Cry toxin injection on larval-pupal and pupal-adult transformation. (A)** Non-viable larval-pupal intermediates showing “incomplete ecdysis” phenotype. Note the presence of both larval and pupal structures. **(B)** Defective adult emerged from the larvae injected with Cry toxin and SEM micrographs of its wing scale morphology. Note the presence of numerous hair-like and under-developed blade-like scales (arrowhead). **(C)** Normal adult emerged from the control larvae and SEM micrographs of normal wing morphology. Note the presence of only lamellar scales with smooth proper transverse and longitudinal ridges (arrowhead).

Injection of Cry toxins to third instar larvae resulted in formation of 20% non-viable larval-pupal intermediate which died and could not molt into pupae (Figure 8A), while 9% of them emerged into defective but viable adults and 5% developed into phenotypically normal adults. The non-viable larval-pupal intermediates exhibited an “incomplete-ecdysis” phenotype. The defective adults that emerged from the toxin injected insects had fairly large but improperly folded and crumbled wings and as a result, the adults could not fly or even mate (Figure 8B, panel 1). On the other hand, only 5% of the control larvae died due to injury during the larval stages while 95% of the control larvae molted normally throughout the larval stages and emerged as normal adults (Figure 8C, panel 1). Scanning electron microscopic analysis of the wing surface morphology of the deformed adult revealed the presence of numerous piliform (hair-like) minute scales in addition to the usual lamellar (blade-like) form of wing scales and most of these lamellar form of scales showed irregular transverse and longitudinal ridges (Figure 8B, panels 2 and 3). On the other hand, the control insects showed the presence of only lamellar scales with smooth inferior lamella and proper transverse and longitudinal ridges (Figure 8C, panels 2 and 3).

## Discussion

Bt-based biopesticides, perhaps the most well-known and widely used biopesticides are now less effective as the number of agricultural insect pests have already developed resistance (Tabashnik et al., 2005; Bravo et al., 2011). Hence an approach that could promise more efficient and eco-friendly control of insect pests in agriculture has become more urgent. No organ or cell other than the midgut epithelial cell has been established as target organ for Cry toxin action in insect pests. However, reports do suggest interaction of Cry toxins with other tissues in the insect body cavity (Arpaia et al., 2000; Cerstians et al., 2001; Hussein et al., 2006). In the larval hemocoel, the major tissues or organs present include the fat body, Malpighian tubule, and salivary gland, where they all play important functions during post-embryonic development. In the present study, hemocoelic injection of four representative Cry toxins i.e., Cry1Aa, Cry1Ab, Cry1Ac, and DOR5 individually to third instar larvae of *A. janata* induced larval mortality, reduced larval growth and gave rise to smaller pupae in a dose-dependent manner, indicating a fairly high insecticidal activity. A dose-dependent increase in larval mortality rate as compared to the control larvae (carrier solvent-injected) clearly suggests that the mortality was the result of toxicity induced by Cry toxins rather than the effects of injury. Since larval mortality was analyzed just 2 days post-injection, we are also convinced that the larvae did not die of starvation, as larvae of this age can normally survive for up to 4–5 days without feeding. Hemocoelic injection to larvae younger than third instar was not possible as they are too small and delicate. DOR5 isolate, which contained *cry1Aa, cry1Ab, cry1C, cry2A, cry2B*, and *vip* genes obviously was more potent than individual recombinant Cry1Aa, Cry1Ab, and Cry1Ac toxins.

The presence of large cytoplasmic cavities in fat body tissue sections suggest degeneration and disintegration of the tissue. Till date, a majority of studies show cytotoxic activity of Cry toxins only against midgut cells of insects. With this study, we report the induction of cell death in fat body tissue of a lepidopteran insect by Cry toxins. In addition, the significant increase in the level of ACP activity primarily in the fat body and not in other tissues indicates involvement of lysosomes in Cry toxin-induced cell death of fat body. During post-embryonic development of holometabolous insects, programmed cell death of fat body follows two major pathways viz. apoptotic and autophagic cell death (Sumithra et al., 2009). During metamorphosis, acidic autophagic vacuoles accumulate in fat body cells and the activity of several lysosomal enzymes including ACP increase and cause the histolysis of larval organs (Ashok and Dutta-Gupta, 1988, 1991; Lee and Baehriecke, 2001). Therefore, it could be that cell death in fat body follows both the pathways during normal development as well as during Cry toxin intoxication, though it may be prematurely induced in the latter case.

The absence of significant change in the ACP activity level in midgut, Malpighian tubule, and salivary gland clearly rules out the involvement of apoptotic and autophagic modes of cell death in these tissues upon Cry toxin injection. However, disorganization of epithelial cells indicates loss of gut tissue structure and damage. This would affect the food storage and digestive roles of the gut and hence disturb the overall supply of nutrients. The chromatin condensation in the nucleus of epithelial cells of Malpighian tubule and a tremendous reduction in the size of salivary gland of the Cry toxin injected larvae are pieces of evidence, suggesting that these tissues are also seriously affected. In a lepidopteran insect like *A. janata*, salivary glands attain maximum size during the last larval instar and pre-pupal stage, and are involved in silk secretion during these stages, which constitute an important physiological event that allows puparia formation for successful larval-pupal transformation. Thus, the decrease in the gland size of the Cry toxin injected larvae and the subsequent insufficient silk secretion during larval-pupal transformation might contribute to abnormal development and metamorphosis seen in the present study. Different larval tissues appeared to respond to Cry toxin injection differently. The fat body being the primary metabolic organ in the larval forms (Price, 1973; Haunerland and Shirk, 1995; Burmester and Scheller, 1999), any damage to this tissue could derail all the ongoing metabolic activities and significantly affect normal development and metamorphosis.

In holometabolous insects, storage proteins, mainly hexamerins are synthesized primarily by fat body of the actively feeding larvae, released into the hemolymph, and later during the non-feeding stage are sequestered back into the fat body again and stored as protein granules, which then serve as the main source of nitrogen and amino acids during pupal-adult metamorphosis and reproduction (Kanost et al., 1990). Contrary to the hemolymph hyperproteinemia reported in the Cry toxin ingested larvae of *S. litura* (Tripathi and Singh, 2002), a significant hypoproteinemia was observed in *A. janata* when Cry toxins were injected into the hemocoel. The significant decline in total protein content of hemolymph associated with a drastic reduction of 80–85 kDa hexamerin proteins to an undetectable level in hemolypmh could indicate inhibition of its synthesis and release, which clearly corroborates with the damage and pathological condition of the fat body seen in toxin injected larvae. Thus, the larval mortality, formation of non-viable larval-pupal intermediates and the emergence of defective adults clearly indicate abnormal development and incomplete metamorphosis which could be the consequences of degeneration of vital larval tissues induced by the toxicity of Cry toxins in the larval hemocoel. There are evidences in literature that when noctuid lepidopteran larvae are fed upon Cry toxin, the toxins are processed in midgut and activated. Further, a small quantity of active toxin was shown to pass through midgut and appear in hemocoel (Pérez-Hedo et al., 2012, 2013; Zhao et al., 2016). The mode of action of Cry toxins in the larval hemocoel is not known but could be different from the one that occurs after oral ingestion. However, binding and pore forming ability of Cry toxin on *in vitro* cultured fat body cells was demonstrated by Cheon et al. (1997), and presence of aminopeptidase in fat body membrane and other non-gut hemocoelic tissue of *A. janata* (Budatha et al., 2007; Ningshen et al., 2013) indicate the presence of Cry toxin binding proteins/receptors which might induce toxicity. In conclusion, the toxicity effects induced by Cry toxins in hemocoel indicate the presence of a specific yet unknown mode of action that possibly targets the tissues and cells present in the insect body cavity. Cry toxin delivery system (e.g., nanoparticle-based) that targets multiple tissues through both oral ingestion and body diffusion/absorption modes predictably would offer a much more effective approach in controlling many economically important pests like *A. janata*.

## Author contributions

Conceived and designed the experiments: AD; Performance of the experiments: TN, VC, and ND; Analysis of the data: AD and TN; Manuscript writing: TN, VC, and ND; Manuscript editing: AD.

### Conflict of interest statement

The authors declare that the research was conducted in the absence of any commercial or financial relationships that could be construed as a potential conflict of interest.

## Supplementary material

Supplementary Figure 1 **Purification of activated Cry toxins for hemocoelic injection**. The activated Cry toxins were purified by gel filtration on Sephadex G-100 column. (**A**) Cry1Aa; Lane M: protein ladder, Lane 1: trypsinized Cry1Aa toxin mixture, remaining lanes: purified activated Cry1Aa toxins. (**B**) Cry1Ab; Lane M: protein ladder, Lane 1: trypsinized Cry1Ab toxin mixture, remaining lanes: purified Cry1Ab toxins. (**C**) Cry1Ac; Lane M: protein ladder; Lane 1: trypsinized Cry1Ac toxin mixture, remaining lanes: purified activated Cry1Ac. (**D**) DOR5; Lane M: protein ladder, Lane 1: discontinuous sucrose density ultracentrifugation prepared DOR5 protoxins, Lane 2: trypsinized DOR5 Cry toxin mixture, remaining lanes: purified activated DOR5 Cry toxins.

Supplementary Figure 2 **Propidium iodide and MTT staining: Fat body tissue was dissected out from toxin injected and control larvae**. The tissue was placed in 500 μl of TC-100 insect culture medium (Sigma-Aldrich, USA) containing trace amounts of streptomycin sulfate. For PI (**A**) staining tissue were washed twice in PBS (pH 7.4) and incubated with 100 μl of 1X PI staining solution (Cat. No. 556463, BD Biosciences, USA) for 15 min. in dark at room temperature. After this 400 μl of 1X binding buffer were added and analyzed under microcope. While for MTT (**B**) 100 μl of staining solution (5 ng/ml) was added and incubated at 25°C with gentle shaking for 30 min in dark. The tissue was then washed twice in PBS (pH 7.4) and mounted on slide and visualized under microcope. The tissue isolated from toxin injected larvae showed the presence of non-viable cells stained reddish pink with PI, which is absent in viable tissue from control. With MTT staining the non-viable fat body cells from toxin injected didn't shows the dark color, while viable tissue from control larvae stained dark purple in color.

## Abbreviations

5E: early fifth instar
5L: late fifth instar
ACP: acid phosphatase
ALP: alkaline phosphatase
APN: aminopeptidase N
BCIP: 5-bromo-4-chloro-3′-indolyl phosphate
BSA: bovine serum albumin
Bt: *Bacillus thuringiensis*
GPI: glycosylphosphatidyl-inositol
IgG: immunoglobulin G
NBT: nitro blue tetrazolium chloride
PP: pre-pupa
SDS-PAGE: sodium dodecyl sulfate polyacrylamide gel electrophoresis.
