# Supplementary figures and images for: Insecticidal Effects of Hemocoelic Delivery of Bacillus thuringiensis Cry Toxins in Achaea janata Larvae
Source: Front Physiol. 2017 May 10;8:289. doi: 10.3389/fphys.2017.00289 (PMC5423935; doi:10.3389/fphys.2017.00289)

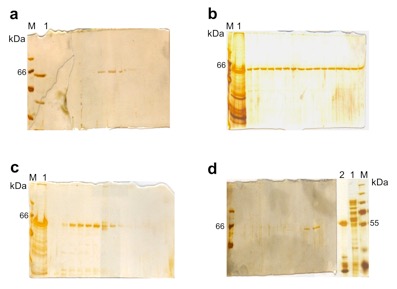

Supplement: Supplementary Figure 1 — Purification of activated Cry toxins for hemocoelic injection. The activated Cry toxins were purified by gel filtration on Sephadex G-100 column. (A) Cry1Aa; Lane M: protein ladder, Lane 1: trypsinized Cry1Aa toxin mixture, remaining lanes: purified activated Cry1Aa toxins. (B) Cry1Ab; Lane M: protein ladder, Lane 1: trypsinized Cry1Ab toxin mixture, remaining lanes: purified Cry1Ab toxins. (C) Cry1Ac; Lane M: protein ladder; Lane 1: trypsinized Cry1Ac toxin mixture, remaining lanes: purified activated Cry1Ac. (D) DOR5; Lane M: protein ladder, Lane 1: discontinuous sucrose density ultracentrifugation prepared DOR5 protoxins, Lane 2: trypsinized DOR5 Cry toxin mixture, remaining lanes: purified activated DOR5 Cry toxins. [file Image1.JPEG]

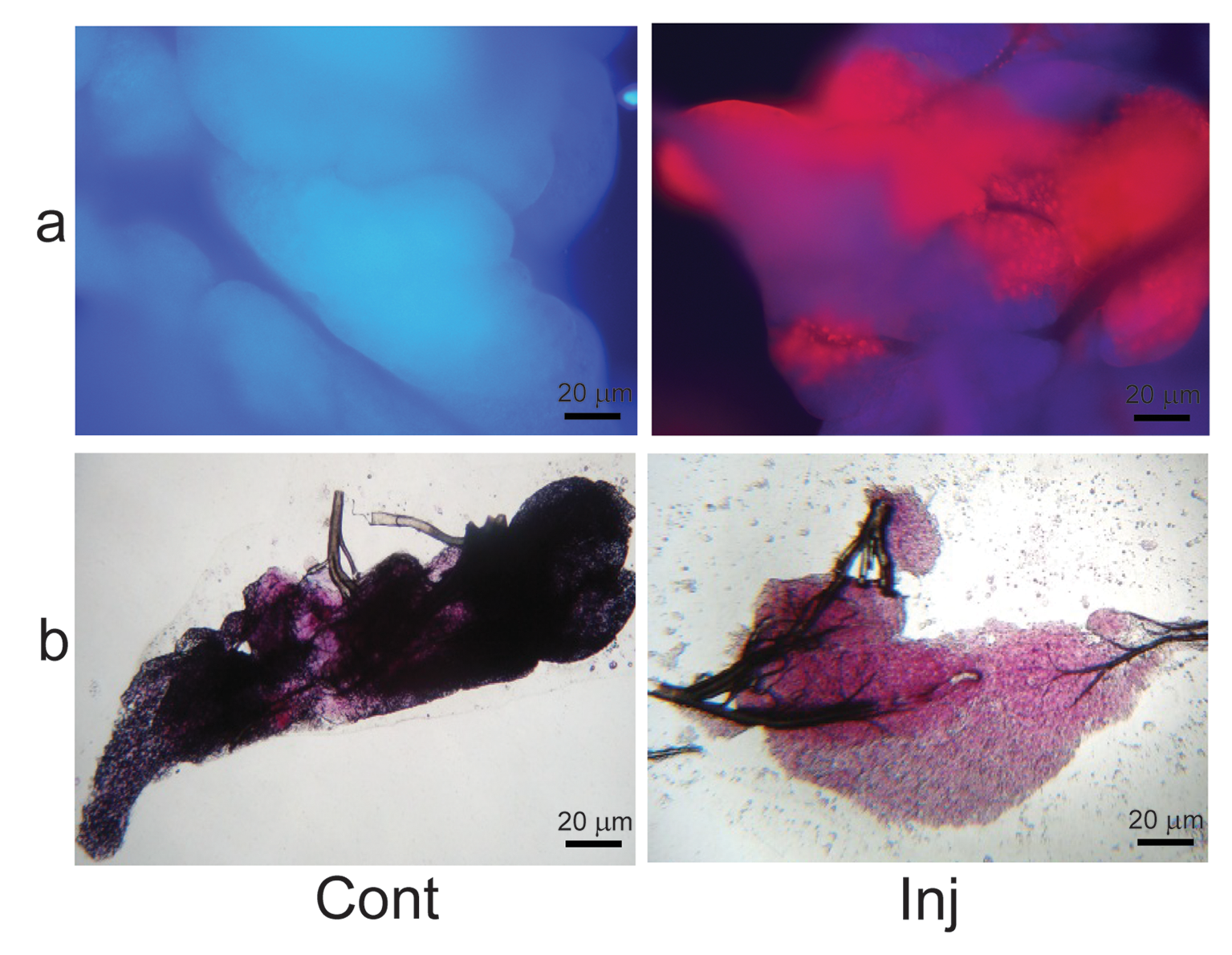

Supplement: Supplementary Figure 2 — Propidium iodide and MTT staining: Fat body tissue was dissected out from toxin injected and control larvae. The tissue was placed in 500 μl of TC-100 insect culture medium (Sigma-Aldrich, USA) containing trace amounts of streptomycin sulfate. For PI (A) staining tissue were washed twice in PBS (pH 7.4) and incubated with 100 μl of 1X PI staining solution (Cat. No. 556463, BD Biosciences, USA) for 15 min. in dark at room temperature. After this 400 μl of 1X binding buffer were added and analyzed under microcope. While for MTT (B) 100 μl of staining solution (5 ng/ml) was added and incubated at 25°C with gentle shaking for 30 min in dark. The tissue was then washed twice in PBS (pH 7.4) and mounted on slide and visualized under microcope. The tissue isolated from toxin injected larvae showed the presence of non-viable cells stained reddish pink with PI, which is absent in viable tissue from control. With MTT staining the non-viable fat body cells from toxin injected didn't shows the dark color, while viable tissue from control larvae stained dark purple in color. [file Image2.TIF]
